# Genome‐scale target capture of mitochondrial and nuclear environmental DNA from water samples

**DOI:** 10.1111/1755-0998.13293

**Published:** 2020-11-27

**Authors:** Mads Reinholdt Jensen, Eva Egelyng Sigsgaard, Shenglin Liu, Andrea Manica, Steffen Sanvig Bach, Michael Møller Hansen, Peter Rask Møller, Philip Francis Thomsen

**Affiliations:** ^1^ Department of Biology Aarhus University Aarhus C Denmark; ^2^ Department of Zoology University of Cambridge Cambridge UK; ^3^ Rambøll Copenhagen S Denmark; ^4^ Natural History Museum of Denmark University of Copenhagen Copenhagen Ø Denmark

**Keywords:** cross‐capture, environmental DNA, mitogenome, nuclear DNA, population genomics, target capture

## Abstract

Environmental DNA (eDNA) provides a promising supplement to traditional sampling methods for population genetic inferences, but current studies have almost entirely focused on short mitochondrial markers. Here, we develop one mitochondrial and one nuclear set of target capture probes for the whale shark (*Rhincodon typus*) and test them on seawater samples collected in Qatar to investigate the potential of target capture for eDNA‐based population studies. The mitochondrial target capture successfully retrieved ~235× (90× − 352× per base position) coverage of the whale shark mitogenome. Using a minor allele frequency of 5%, we find 29 variable sites throughout the mitogenome, indicative of at least five contributing individuals. We also retrieved numerous mitochondrial reads from an abundant nontarget species, mackerel tuna (*Euthynnus affinis*), showing a clear relationship between sequence similarity to the capture probes and the number of captured reads. The nuclear target capture probes retrieved only a few reads and polymorphic variants from the whale shark, but we successfully obtained millions of reads and thousands of polymorphic variants with different allele frequencies from *E. affinis*. We demonstrate that target capture of complete mitochondrial genomes and thousands of nuclear loci is possible from aquatic eDNA samples. Our results highlight that careful probe design, taking into account the range of divergence between target and nontarget sequences as well as presence of nontarget species at the sampling site, is crucial to consider. eDNA sampling coupled with target capture approaches provide an efficient means with which to retrieve population genomic data from aggregating and spawning aquatic species.

## 1 INTRODUCTION

Population genomic analyses have become an efficient way of studying population structure, demography, selection and dispersal in numerous species. Advances in DNA sequencing technology have enabled researchers to sequence hundreds of whole‐genomes with reasonable effort and for reasonable costs (Schwarze et al., 2020). While these advances have improved opportunities for studying natural populations in the wild, many species, especially large marine species, remain difficult to sample efficiently and noninvasively. Furthermore, obtaining permits (e.g., CITES, export, import) for sampling tissue can be cumbersome and time‐demanding, and international transport of animal samples can be disruptive to project logistics.

Environmental DNA (eDNA) sampling has in recent years emerged as a powerful way of determining the species composition of contemporary ecosystems (Stat et al., 2017; Thomsen, Kielgast, Iversen, Møller, et al., 2012; Thomsen, Kielgast, Iversen, Wiuf, et al., 2012; Thomsen & Willerslev, 2015; Zinger et al., 2019). eDNA methods can be a cheaper alternative to traditional sampling methods (Evans et al., 2017; Mahon et al., 2013), they offer a noninvasive approach compared to tissue samples (Sigsgaard et al., 2017), and in the marine environment, the approach has proven useful in detecting both elusive (e.g., Boussarie et al., 2018; Mauvisseau et al., 2017), cryptic (Agersnap et al., 2017; Port et al., 2016), rare (Sigsgaard et al., 2017; Weltz et al., 2017) and invasive species (e.g., von Ammon et al., 2019; Miralles et al., 2016; Wood et al., 2017).

While eDNA studies have already made large contributions to biodiversity research at the species level, the potential for eDNA methods in retrieving population genetic information has only just begun to be explored (Adams et al., 2019; Sigsgaard et al., 2020).

It was recently shown that mitochondrial control region (CR) haplotype frequencies found in tissue samples of whale shark (*Rhincodon typus*) in Qatar were mirrored in eDNA metabarcoding (Taberlet et al., 2012) of seawater samples from the same study site (Sigsgaard et al., 2017). Similar results were later obtained by Parsons et al. (2018) for harbour porpoises *Phocoena phocoena* and by Baker et al. (2018) for killer whales *Orcinus orca*.

However, the fragmented nature of eDNA, along with the limited read lengths available using Illumina sequencing, have restricted eDNA metabarcoding to focus on relatively short amplifiable regions of the mitochondrial DNA (mtDNA). While a short variable marker can successfully provide haplotype information (Sigsgaard et al., 2017; Turon et al., 2020), it provides limited resolution. Future population genetic eDNA studies would therefore benefit from a greater coverage of mtDNA variation, and ideally from incorporating nuclear DNA (nuDNA) markers. As all mtDNA segments are physically linked, they do not provide independent information. Hence, analysis of nuclear DNA should, if possible, be the preferred option, also to avoid bias due to mtDNA being maternally inherited.

For eDNA research to produce more powerful population genetic inferences, the potential for analysing a greater part of the mitogenome and to include multiple markers of nuDNA therefore needs to be investigated. Because environmental water samples contain DNA from various nontarget species, for example more than 99% when working with eukaryotes as the target group (Stat et al., 2017), target capture approaches are a promising alternative to shotgun sequencing (Sigsgaard et al., 2020). Target enrichment via DNA hybridization capture (“target capture”) (Gnirke et al., 2009), is a well‐tested method for obtaining DNA data from samples with high nontarget content. In short, custom biotinylated RNA baits hybridize with complementary DNA sequences from the sample, and nonhybridized sequences are washed away, ultimately enriching the sample for the target DNA, while avoiding issues related to PCR bias (Polz & Cavanaugh, 1998).

Target capture is well known from ancient DNA (aDNA) research, for enriching endogenous components of DNA from samples of, for example, bone or hair (Carpenter et al., 2013; Cruz‐Dávalos et al., 2017; Paijmans et al., 2016). Recently, the approach has also been implemented on ancient (Slon et al., 2017) and contemporary (e.g., Seeber et al., 2019) eDNA samples. Seeber et al. (2019) used target capture of eDNA from water holes to elucidate contemporary terrestrial mammal species richness, and Mariac et al. (2018) designed a single probe targeting cytochrome *c* oxidase subunit 1 (COI) as a potential alternative approach for species detection in ichthyoplankton swarms. Furthermore, single taxon capture probes have been developed for contemporary eDNA from water samples to evaluate species detection efficiency (Wilcox et al., 2018). Pinfield et al. (2019) applied whole‐genome enrichment capture with RNA baits followed by subsequent shotgun sequencing of eDNA samples, but not enough killer whale DNA was retrieved to conduct population genetic analyses and infer a potential source population.

The whale shark feeding aggregation studied by Sigsgaard et al. (2017) provided ideal conditions for testing a population‐level eDNA approach, as many individuals are concentrated in a small area, and reference mtDNA sequences were available from both Qatar and other parts of the world. Recent efforts into sequencing the whale shark genome (Hara et al., 2018; Read et al., 2017; Weber et al., 2020) have now enabled the design of genome‐wide capture probes for the species and mapping of potential whale shark sequences obtained from eDNA target capture.

We developed and tested one mitochondrial and one nuclear set of target capture probes for the whale shark to investigate the potential for extracting population genomic data from eDNA samples. We successfully retrieved (a) eDNA reads spanning the entire mitochondrial genome of whale sharks, which furthermore matched previously known haplotypes, and (b) nuclear reads covering multiple loci in the whale shark genome. As an interesting addition, we also retrieved a large amount of reads from the fish species mackerel tuna (or kawakawa) (*Euthynnus affinis*), the eggs of which are the probable cause of the whale shark aggregations in the area (Robinson et al., 2013). These data enabled us to investigate patterns of mitochondrial sequence‐to‐probe similarity in relation to coverage obtained and to estimate allele frequencies at multiple loci from nuclear reads of *E. affinis*.

## 2 MATERIALS AND METHODS

### 2.1 Sample collection, extraction and initial testing

Two 1‐L water samples were filtered through sterile 0.22‐µm Sterivex‐GP filters (Merck Life Science) directly from a boat at the Al Shaheen oil field in Qatar on September 1, 2016. The two samples were collected from surface water in the middle of an aggregation of >50 whale sharks visible by eye. We did not investigate the presence of other species at the sampling site, but the whale sharks are thought to aggregate in these waters to feed on the eggs of spawning *Euthynnus affinis* (Robinson et al., 2013; Sigsgaard et al., 2017), which we thus expected to be highly abundant. The filters were immediately put on ice and stored at −20°C until DNA extraction. Separate DNA extractions were carried out for the two samples using the DNeasy Blood & Tissue kit (Qiagen). The manufacturer's protocol was slightly modified, using four times more AL buffer and proteinase K and 3 hr of incubation. Samples were initially screened for whale shark eDNA with two sets of species‐specific TaqMan qPCR systems (TAG Copenhagen) (Text A in the Appendix S1).

### 2.2 Development of nuclear target capture system

Using the published whale shark genome (Read et al., 2017), we designed a bait system (59,941 probes total, targeting ~0.1% of the genome) aimed at enriching primarily for nuclear intron fragments of whale shark DNA. We expect introns to be more variable, as they are subject to fewer functional constraints, and thus to provide more information for population genetic inferences (Li, 1997). However, a small proportion of exon baits were also included. The nuclear bait set costs ~€160 per reaction including the design process (but not including library kit and indexing), with a minimum of 16 reactions. For details on the nuclear capture design see Text B in the Appendix S1.

### 2.3 Development of the mitochondrial target capture systems

A “myBaits Mito” kit (Catalogue no. 303096) was designed by Arbor Biosciences from the mitogenome of the Taiwanese whale shark specimen sequenced by Read et al. (2017) (NCBI accession no. NC_023455.1). A probe system with 80‐bp probes and 4× tiling was created to capture the entire mitogenome. We specifically kept nuclear and mitochondrial target capture separate, as the multicopy nature of organellar genomes is known to cause sequencing output to be dominated by organellar DNA, with minimal amounts of potential nuclear DNA being captured and sequenced (Andermann et al., 2020; Falk et al., 2012). The mitochondrial bait set costs ~€30 per reaction including the design process (but not including library kit and indexing), when purchasing 96 reactions at a time.

### 2.4 Library preparation and sequencing

Fragment sizes of the raw eDNA extracts were initially visualized on a 4200 TapeStation (Agilent). The two samples were then pooled into one in equal volumes to ensure sufficient starting material, and thus now represent a single sample of 2 L of filtered water. The pooled sample was sonicated on an S220 Focused‐Ultrasonicator (Covaris), aiming for a fragment size of ~250 bp. A single library was built using the Accel‐NGS 2S Hyb DNA Library Kit (Cat. No. 23096) (Swift Biosciences) and used as input for both the mitochondrial and nuclear capture. We used 13.33 µl eDNA template (~200 ng total) in the library preparation, and the capture reactions were carried out following the supplied protocol, running seven precapture PCR cycles, 48 hr of hybridization at 65°C, and 14 post‐capture PCR cycles. The final, enriched products from the mitochondrial and nuclear capture were then purified and sequenced (301 bp paired‐end) in two separate runs on a MiSeq (Illumina) at the Department of Biology, Aarhus University.

### 2.5 Mitochondrial whale shark capture

Mitochondrial paired‐end reads were filtered and collapsed using adapterremoval version 2 (Schubert et al., 2016), specifying a minimum Phred quality score of 20 and a minimum read length of 40 bp. Reads were first searched against the published whale shark mitogenome using blastn and with only a ≥70% sequence similarity criterion, acknowledging the highly variable D‐loop region (Brown et al., 1986). The retained reads were then searched against the entire nucleotide database in GenBank. As this database contains mitochondrial sequences from multiple whale shark individuals, only reads with whale shark as best blastn hit and a minimum sequence similarity of 98% were retained after dereplication of identical sequences with vsearch‐2.14.2 (Rognes et al., 2016). All retained mitochondrial reads were imported into geneious (Kearse et al., 2012), where data were visualized and all subsequent analyses were carried out. All plots were made using the R package “ggplot2” (Wickham, 2016).

### 2.6 Mitochondrial nontarget capture

As cocaptured nontarget reads could be of interest for evaluating capture efficiency, we performed a blastn search on all quality filtered reads for the mitochondrial capture and extracted all sequences from the three species with higher numbers of hits than whale shark, that is *E. affinis*, skipjack tuna (*Katsuwonus pelamis*) and striped bonito (*Sarda orientalis*). We mapped these sequences to the mitogenomes of their respective species and inspected both coverage distribution and variable sites, with a minor allele frequency (MAF) filter of 5%.

In order to investigate capture efficiency, we aligned mitogenomes of the three scombrid species (accession nos. *E. affinis* NC_025934, *K. pelamis* JN086155, and *S. orientalis* AP012949) to the whale shark mitogenome (accession no NC_023455). We calculated similarity between the mitogenomes of each scombrid species to the whale shark using a sliding mean across the entire mitogenome. For every base pair, we included the 5 bp before and after that position to determine similarity (11 bp in total). As aligning these sequences inevitably led to gaps in the alignment, the alignment was longer than the actual mitogenome. In order to relate sequence similarity to the sequencing depth, we therefore disregarded gaps inserted in the scombrid mitogenomes for both similarity and coverage scores (Liu et al., 2019).

### 2.7 Nuclear whale shark capture

Nuclear paired‐end reads were filtered and collapsed exactly as the mitochondrial reads. The reads were blastn searched against the whale shark genome, and all hits with ≥97% match were retained. These reads were then blastn searched against four other chondrichthyan genomes (Australian ghostshark *Callorhinchus milli*, little skate *Leucoraja erinacea*, cloudy catshark *Scyliorhinus torazame*, and brownbanded bamboo shark *Chiloscyllium punctatum*) used for probe design (Text B in the Appendix S1), as well as against the entire nucleotide database downloaded from GenBank (downloaded September 2019). All reads with a highest or tied match to the whale shark genome were retained after dereplication, and subsequently mapped to the whale shark genome using bwa‐0.7.17 (Li & Durbin, 2009). The mapped reads were filtered for a minimum mapping quality (MAPQ) of 20 using samtools‐1.9 (Li et al., 2009). Variants were called using samtools and bcftools, and filtering of nuclear variants was done using snpsift‐4.3t (Cingolani et al., 2012).

### 2.8 Nuclear nontarget capture

As a large proportion of nuclear reads were assumed to stem from *E*. *affinis*, and as there is no complete nuclear genome available for this species, reads were mapped to the genome of the confamilial species Atlantic bluefin tuna (*Thunnus thynnus*) (accession no. GCA_003231725.1) using bwa‐0.7.17 (Li & Durbin, 2009) and samtools‐1.9 (Li et al., 2009). Here, we implicitly assume that a high abundance of mitochondrial reads from *E. affinis* would correlate with a similarly high abundance of nuclear reads within a sample. The genetic distance between *E. affinis* and *T. thynnus* is about 11.3% (inferred from aligning mitochondrial genomes, accession nos. *E. affinis* NC_025934 and *T. thynnus* AP006034). Reads were dereplicated and mapped and variants were called and filtered exactly like the whale shark reads above.

## 3 RESULTS

### 3.1 Mitochondrial capture of whale shark eDNA

**FIGURE 1:**
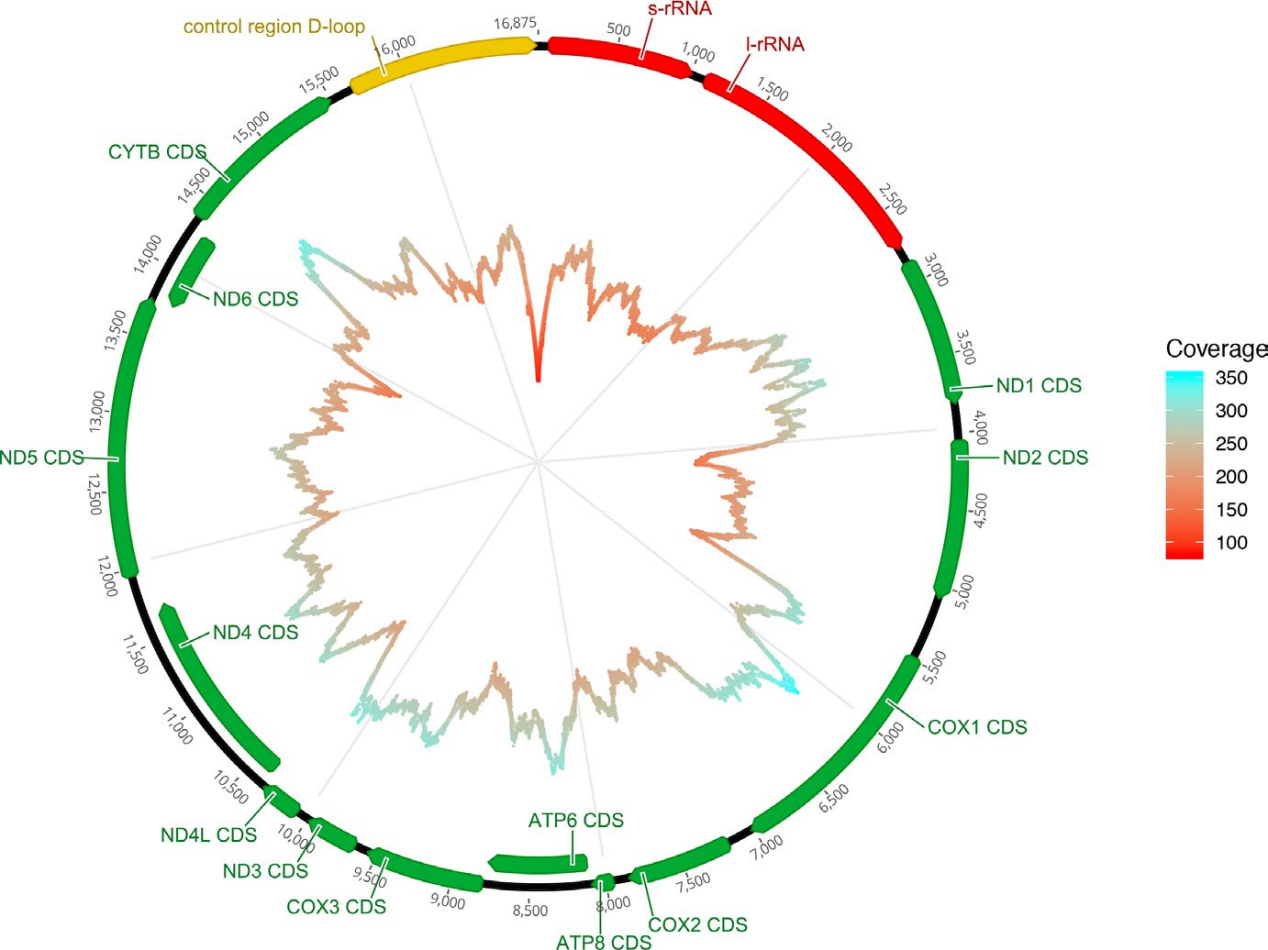
A graphic and clockwise overview of the coverage obtained from mapping putative whale shark reads from the mitochondrial capture to the whale shark mitogenome (accession no. NC_023455.1). The innermost line depicts individual base pair coverage (coloured from low [red] to high [light blue]), and the outer circle represents the annotated whale shark mitogenome for reference [Colour figure can be viewed at wileyonlinelibrary.com]

Both samples tested in the initial qPCRs were confirmed to contain mitochondrial whale shark DNA (C_t_‐values of replicates: 28.63, 29.07, and 31.42, 31.45, respectively). MiSeq data for the pooled sample provided an initial 14.7 million reads passing the quality and length requirements. After filtering to ensure whale shark was the best hit with at least 98% sequence similarity, 27,875 reads were retained (~0.19% of all reads on target). After dereplication, a total of 16,486 unique reads were retained, of which 16,474 mapped to the whale shark mitogenome (NC_023455). With an average read length of ~240 bp (min: 63 bp, max: 589 bp; Figure S1) and a very even distribution of reads across the mitogenome, we thus obtained a ~235× (min: 90×, max: 352×) coverage per base position of the whale shark mitogenome (Figure 1).

The genetic variation found in the data set reflected known haplotype variation from Qatar, as we found four sequences with complete coverage of three different D‐loop haplotypes previously found by Sigsgaard et al. (2017) using both tissue samples and eDNA samples (2 × DL1‐A, 1 × DL1‐C and 1 × DL1‐D). We furthermore observed the single nucleotide polymorphisms (SNPs) responsible for the haplotypes DL1‐B and DL1‐E, although we did not recover any single sequences spanning the entire region of these haplotypes. Furthermore, when applying a 5% MAF filter, we found a total of 29 variable sites throughout the mitogenome (Table 1). In general, these variants corresponded well with previously known variants in the D‐loop region based on tissue samples, but we also recovered eight putatively new variants from gene regions that have not been sequenced exhaustively for *Rhincodon typus*.

**Table 1:** Overview of mitochondrial variants when mapping putative whale shark reads to the whale shark mitogenome used for bait design (accession no. NC_023455) with a 5% minor allele frequency requirement

Variant no.	Loc	Gene	Nucl. change	Mut type	AA change	CDS position	Codon change	Cov	Prot effect	Allele freq (%)	KV
1	359	12S	G → A	TI				198		6.1	–
2	768	12S	A → G	TI				185		63.8	+
3	4,088	ND2	G → A	TI	G → S	40	GGT → AGT	175	Sub	6.9	+
4	4,588	ND2	A → G	TI		540	GCA → GCG	210	None	13.3	–
5	5,338	OL	A → G	TI				175		60.6	+
6	8,050	ATP8	T → C	TI	F → S	98	TTC → TCC	260	Sub	6.2	–
7	8,509	ATP6	T → C	TI		399	GGT → GGC	269	None	61.3	+
8	11,064	ND4	T → C	TI		705	ATT → ATC	275	None	57.5	+
9	11,848	tRNA‐ser	T → C	TI				252		7.5	–
10	12,117	ND5	T → C	TI		169	TTA → CTA	240	None	6.7	–
11	13,637	ND5	G → A	TI		1689	GAG → GAA	212	None	63.7	+
12	14,654	Cyt B	T → C	TI		288	TAT → TAC	322	None	14.9	–
13	14,870	Cyt B	C → A	TV		504	GGC → GGA	244	None	32.4	–
14	15,539	tRNA‐Thr	T → C	TI				200		56.5	+
15	15,707	D‐loop	A → G	TI				215		35.3	+
16	15,772	D‐loop	T → C	TI				180		59.4	+
17	15,791	D‐loop	T → C	TI				196		7.7	+
18	15,879	D‐loop	G → A	TI				189		12.7	+
19	15,898	D‐loop	+ATGTACGTCA	INS				187		12.8	+
20	15,919	D‐loop	A → G	TI				179		89.9	+
21	15,922	D‐loop	C → T	TI				179		21.2	+
22	15,983	D‐loop	T → C	TI				174		35.6	+
23	16,002	D‐loop	T → C	TI				182		8.8	+
24	16,034	D‐loop	C → T	TI				197		41.6	+
25	16,061	D‐loop	+ATATGATCTTCCACATT	INS				203		17.2	+
26	16,244	D‐loop	T → C	TI				185		13.0	+
27	16,259	D‐loop	A → G	TI				196		7.7	+
28	16,443	D‐loop	C → T	TI				225		35.1	+
29	16,724	D‐loop	(A)10 → (A)11	INS				155		11.6	–

Note

Loc: location of the variant. Mut type: type of mutation, either transition (TI), transversion (TV) or insertion (INS). AA change: amino acid change. CDS position: location of the variant within the coding sequence. Cov: coverage. Prot effect: protein effect, either substitution (Sub) or none. KV: known variant from sequenced tissue samples of whale sharks deposited in GenBank (+) or unknown variant (–).

### 3.2 Mitochondrial capture of nontarget eDNA

A large majority of the quality filtered sequencing reads matched *Euthynnus affinis* as best blast hit. A much lower number of reads matched *Katsuwonus pelamis* and *Sarda orientalis*, and these hits primarily occurred in conserved regions with lower taxonomic resolution. Importantly, when disregarding family‐level hits with <100 sequences, only about 0.075% of the mitochondrial reads with 100% hits in GenBank were hits to bacteria and algae (Table S1). Applying similar filters (98% match criterion, best hit) as to the whale shark sequences described above, we retained 2,441,828 unique *E. affinis* reads, 122,582 *K. pelamis* reads and 12,395 *S. orientalis* reads. When mapping these dereplicated reads to the species’ respective mitogenomes, 2,441,523, 122,563 and 12,395 reads were mapped, respectively.

**FIGURE 2:**
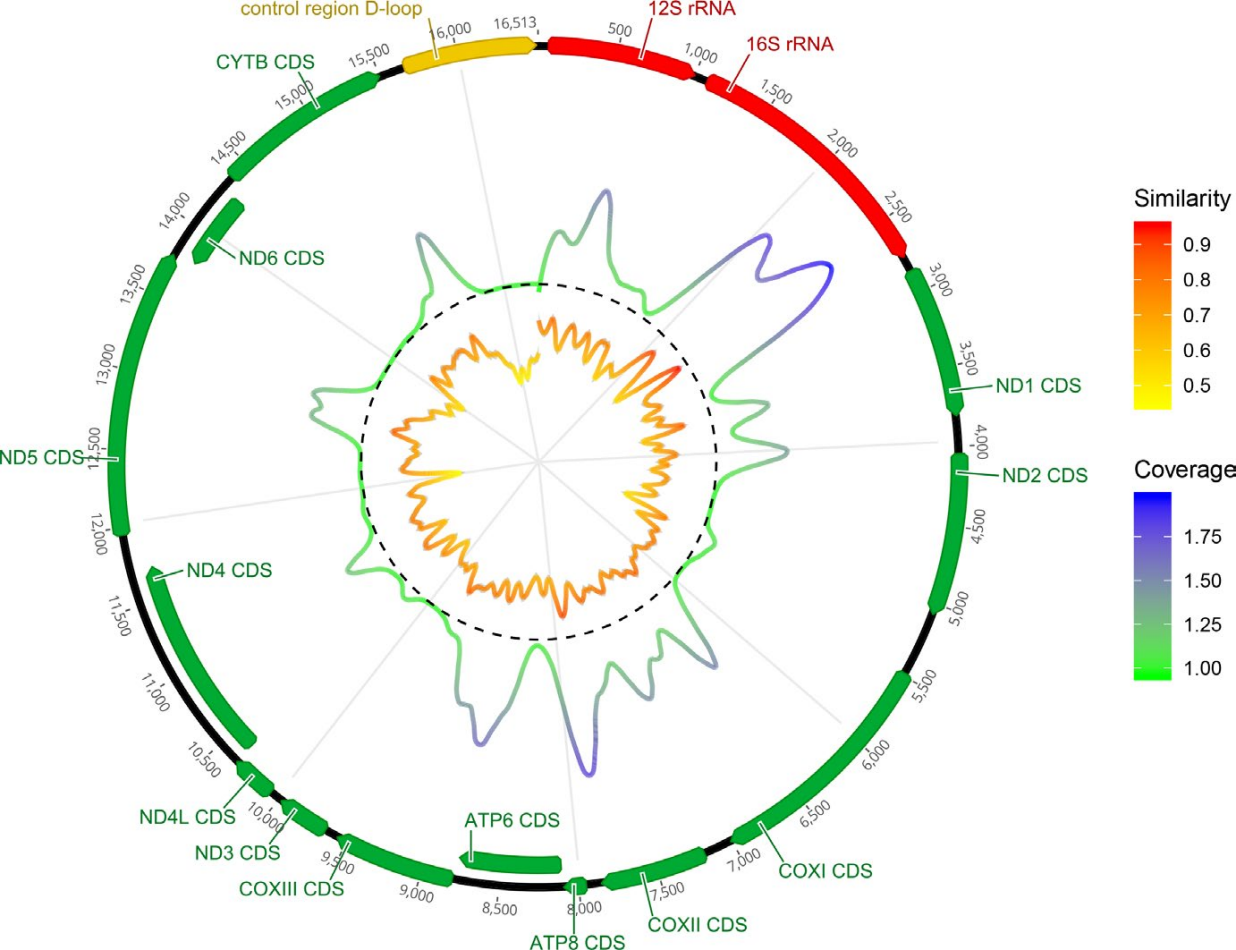
A graphic and clockwise overview of the similarity between *Euthynnus affinis* and *Rhincodon typus* mitogenomes as well as coverage obtained from mapping putative *E. affinis* reads from the mitochondrial capture to the *E. affinis* mitogenome (accession no. NC_025934). The innermost line depicts a “sliding mean” similarity between the two mitogenomes (coloured from low [yellow] to high [red]). The dashed line (*y* = 1) represents both the maximum similarity possible (identical) and the minimum coverage. Transposed coverage is presented on top of the dashed line, where the value 1 reflects zero coverage and 2 reflects maximum coverage (223,888×). The outer circle represents the annotated mitogenome of *E. affinis* [Colour figure can be viewed at wileyonlinelibrary.com]

While hits to *E. affinis* (accession no. NC_025934) covered almost the entire mitogenome (except 2 bp in the D‐loop), we saw a large discrepancy in individual base pair coverage, spanning from zero to 233,808 × coverage. Using the similarity measures calculated between *E. affinis* and *R. typus*, we find an average sequence divergence of 29.3% between the two mitogenomes (disregarding gaps, similarity range of 0 to 1 based on 11 bp). Comparing sequence similarity between the *R. typus* and *E. affinis* mitogenomes with the target capture coverage of *E. affinis*, linear regression (*y* = 0.31*x* − 0.032, adjusted *R* ^2^ = .058) indicates a statistically significant (*p* < .001) pattern of higher similarity resulting in higher coverage (Figures 2 and S2). Thus, more conserved gene regions such as the latter parts of the 16S rRNA gene are captured at a higher rate than, for example. the D‐loop. Table S2 provides details on mitochondrial variants from *E. affinis* reads and a comparison with sequenced tissue samples.

### 3.3 Nuclear capture of whale shark eDNA

**FIGURE 3:**
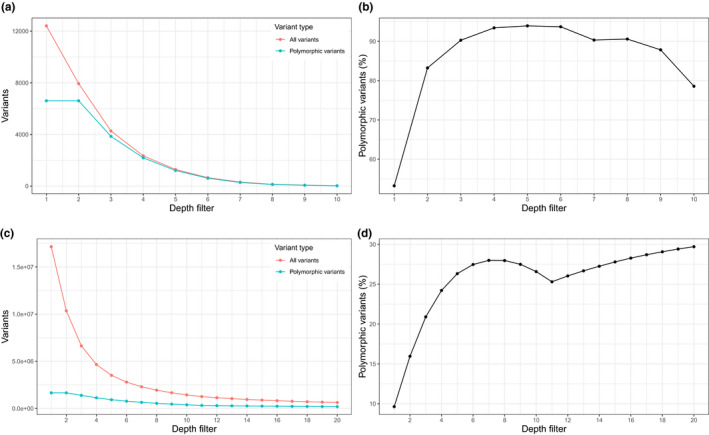
Overview of variants found using nuclear capture probes when mapping putative whale shark reads to the whale shark genome (accession no. GCA_001642345.2) (a,b) and when mapping quality‐filtered, dereplicated raw reads to the *Thunnus thynnus* genome (accession no. GCA_003231725.1) (c,d). (a,c) The number of variant sites retained as depth filter (i.e., minimum coverage required for a variant to be retained) increases for both “all variants” and “polymorphic variants” (variants where both the reference allele and another allele are present in the data). (b,d) The relative percentage of polymorphic variants compared to total variants across the same depth filter gradient [Colour figure can be viewed at wileyonlinelibrary.com]

Both samples tested in the initial qPCRs were confirmed to contain nuclear whale shark DNA (C_t_‐values for triplicates: 36.71, 36.66, 36.30 and 40.25, 41.09, 40.09, respectively). Filtered MiSeq data for the pooled sample provided an initial 16.3 million reads passing the quality and length requirements. After ensuring highest or equally high match to the whale shark genome, 89,882 reads were retained (0.55% of all reads) with an average Phred quality score > 37. After dereplication and mapping to the whale shark genome, 48,433 reads were retained with an average read length of ~262 bp (min: 47 bp, max: 590 bp; Figure S3). For details on coverage distribution, see Figure S4. From the mapped reads, we found 12,411 raw variants, but most of the variants only had 1–2 × coverage (Figure 3) and a large proportion (46.76%) of the raw variants were simply monomorphic deviations from the reference genome. Based on a rough estimate of an expected PCR error rate of 1.98% (21 PCR cycles in total, 252.6 bp average sequence length, KAPA HI‐FI polymerase with an estimated one error per 3.6 × 10^6^ nucleotides incorporated) and a sequencing error rate of 0.0002 (Phred = 37), we would expect that about half (~50.87%) of these raw variants represent errors derived from sequencing and PCR. Increasing the depth filter (i.e., minimum coverage required for a variant to be retained) to 10× coverage resulted in 22 polymorphic variants retained (Table 2), the majority of which were only represented by a single sequence deviating from the reference sequence.

**Table 2:** Overview of nuclear variants retained with a coverage of ≥10 when mapping to the whale shark genome used for bait design (accession no. GCA_001642345.2)

Variant no.	Contig	Cov	Position of variant	Nucl. change	Mut type	Exon	Intron	Within probe range (±80 bp)	MAF	Gene
1	NW_018027618.1	10	81,300	T → A	TV		+	+	0.10	
2	NW_018028177.1	10	361,879	G → A	TI	+		+	0.10	arhgap31
3	NW_018028334.1	10	259,900	C → T	TI		+	+	0.10	
4	NW_018028334.1	10	259,937	A → G	TI		+	+	0.10	
5	NW_018028334.1	10	259,952	C → T	TI		+	+	0.10	
6	NW_018030239.1	10	300,687	A → G	TI		+	+	0.10	
7	NW_018030239.1	10	300,695	A → T	TI		+	+	0.10	
8	NW_018031751.1	10	272,654	AGTTTCTGT → AGT	DEL		+	+	0.30	
9	NW_018031751.1	10	272,838	G → T	TV		+	+	0.10	
10	NW_018032444.1	10	219,962	T → C	TI		+	+	0.10	
11	NW_018033032.1	10	57,538	C → T	TI		+	+	0.10	
12	NW_018034852.1	10	57,766	A → G	TI			–	0.10	
13	NW_018035951.1	10	70,329	C → T	TI		+	+	0.10	
14	NW_018048269.1	10	39,955	A → G	TI		+	+	0.10	
15	NW_018049874.1	64	2,967	A → G	TI			–	0.05‬	
16	NW_018055946.1	26	795,896	T → C	TI		+	–	0.23	
17	NW_018055946.1	28	795,897	A → G	TI		+	–	0.21	
18	NW_018056210.1	10	348,679	A → G	TI		+	+	0.10	
19	NW_018061029.1	10	213,227	A → G	TI		+	+	0.10	
20	NW_018067024.1	10	123,091	C → G	TV		+	+	0.10	
21	NW_018071985.1	10	326,258	T → A	TV		+	+	0.10	
22	NW_018071985.1	10	326,259	G → C	TV		+	+	0.10	

Note

Mut type: type of mutation, either transition (TI), transversion (TV) or deletion (DEL). Exon: variant occurs in exonic region (+). Intron: variant occurs in intronic region (+).

Abbreviations: Cov, coverage; MAF, minor allele frequency.

The majority of the variants retained (82%) reside in regions targeted by the nuclear capture system. Furthermore, our approach was also successful in capturing both intronic and exonic regions (Text C in the Appendix S1), although only a single exonic variant was retained with a 10× coverage filter.

### 3.4 Nuclear capture of *Euthynnus affinis* eDNA

**FIGURE 4:**
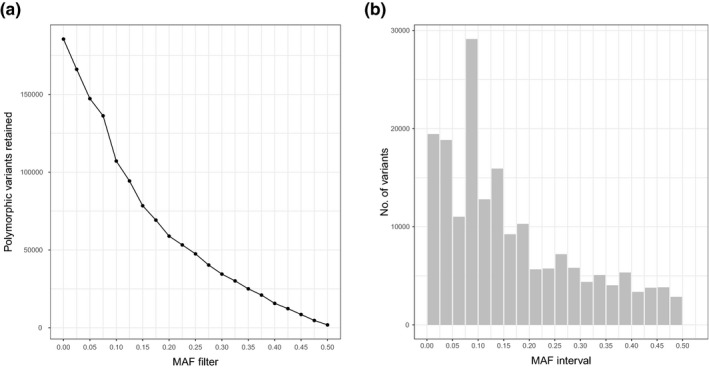
Overview of variants retained with nuclear capture probes when mapping all quality‐filtered, dereplicated raw reads to the *Thunnus thynnus* genome (accession no. GCA_003231725.1) with a constant depth filter (i.e., minimum coverage required for a variant to be retained) of 20 (see Figure 3 “polymorphic variants”) and an increasing minor allele frequency filter. (a) Polymorphic variants retained with increasing MAF‐filter (0–0.5). (b) Histogram of the number of variants lost with each MAF‐filter increment, resembling a minor allele frequency spectrum based on read counts. Each bar thus represents all variants that fall within an interval of allele frequencies with an increment of 0.025 per bar, starting from 0 to 0.025 [Colour figure can be viewed at wileyonlinelibrary.com]

After quality filtering and dereplication of raw reads, 10,062,899 reads remained. Of these, 7,156,494 (71.1%) of the reads were successfully mapped to the *Thunnus thynnus* genome. Variant filtering resulted in 17,151,020 raw variants of which 1,651,793 were polymorphic (Figure 3). As these nuclear reads were mapped to a genome from *T. thynnus* rather than *E. affinis*, we expect most of the monomorphic variants to simply represent differences between the two tuna genomes. Adding a depth filter removed a large proportion of the polymorphic variants (Figure 3), as 88.76% of the polymorphic variants were lost with a depth filter of 20, although it should provide a higher confidence in allele frequencies than the depth filter of 10 for whale sharks. Inspecting the MAF distribution of the remaining variants, however, shows a broad diversity of allele frequencies across the different variants, with a relatively larger proportion of the variants appearing in low frequencies (Figure 4).

## 4 DISCUSSION

### 4.1 Capture efficiency

Despite the complex DNA composition of environmental samples, we here demonstrate how target capture protocols can enrich eDNA samples for desired DNA fragments. In contrast to direct shotgun sequencing of eDNA from water samples (where bacterial input dominates the sequencing output Cowart et al., 2018; Stat et al., 2017), we here generated a data set with minimal bacterial dominance (see Table S1). While we initially intended to focus solely on optimizing the sequencing output for whale shark sequences, the target capture turned out not to be strictly species‐specific. This is in accordance with a previous study on chondrichthyans with myBaits probes, which have reported up to 39% divergence between baits and captured targets (Li et al., 2013), although their protocol was optimized for divergent homologue sequence capture through a touchdown gene capture (Mason et al., 2011). In our study, the vast majority of the sequencing data for both capture protocols were from *Euthynnus affinis*, and although we do observe a pattern of high similarity leading to higher coverage, DNA input from bony fishes cannot be avoided, especially when conserved regions are targeted. We kept the incubation temperature to the maximum recommended (65°C) throughout the capture process, and we would thus expect lower capture rates of highly divergent sequences. However, with our estimated average sequence divergence between the *E. affinis* and *Rhincodon typus* mitogenomes of 29.3%, and the large differences in between‐species similarity across the mitogenome (Figure 2), some level of nontarget capture is inevitable. While the nuclear target capture retrieved a larger relative proportion of whale shark sequences than the mitochondrial capture (0.55% vs. 0.19% of reads), nontarget capture is probably also unavoidable for nuclear data, especially if probes are designed for exonic regions. Nevertheless, our results indicate that these nontarget data can be highly informative.

While designing capture probes enables us to retrieve far more targeted genetic information than metabarcoding and direct shotgun sequencing approaches would permit on water samples targeting single species, it is also an expensive solution. However, it is highly scalable once the probes have been developed, and the price will drop markedly when reordering previously designed probe sets as well as larger quantities. It would be interesting to compare the resulting fold‐increase of targeted capture approaches with direct shotgun sequencing on the same samples in relation to the price, in order to fully undertand its merits for environmental samples.

### 4.2 Mitochondrial capture

The results from mitochondrial capture highlight the strong applicability of this approach, as we were able to obtain a ~235× coverage mitogenome of the whale shark. We acquired data containing a large amount of both known and unknown variation across the mitogenome, with the variation in the D‐loop region being in concordance with previous studies on the same whale shark aggregation (Sigsgaard et al., 2017). The finding of three complete previously known D‐loop haplotypes, as well as two SNPs indicative of two additional haplotypes, provides support for the whale shark origin of the captured sequences, and we can conservatively suggest that at least five different whale shark individuals contributed mtDNA to the sequenced water sample. To discern between rare variants and sequencing errors, we applied an MAF‐filter and illustrated how this approach can be used as a variant exploration tool for eDNA sequences across the whale shark mitogenome. While the read lengths obtained from target capture are limited when using current high‐accuracy sequencing platforms, we obtained reads long enough to confirm previously known haplotypes. However, a transition to long‐read sequencing on third‐generation sequencing platforms would increase resolution significantly, assuming eDNA samples are not overly degraded regarding sequence length and that the error rates of these platforms improve substantially.

Importantly, a great advantage of the target capture approach is that high‐coverage sequence data from entire mitogenomes can be retrieved, instead of relying on a single species‐specific metabarcoding marker, as is the current standard (Baker et al., 2018; Parsons et al., 2018; Sigsgaard et al., 2017). Obtaining accurate estimates of the number of individuals contributing to an eDNA sample based on mitogenomic data is at present unfeasible (see Sigsgaard et al., 2020 for a discussion of the challenges associated with identification of individuals), and we here limit ourselves to a conservative minimum estimate based on the number of haplotypes found. To guide further research on this issue, we suggest three approaches which could be vital for understanding the link between eDNA population genetic diversity and the number of contributing individuals: (a) sequencing mock samples with varying numbers of individuals in a pooled sequencing approach in parallel with eDNA samples; (b) a systematic experimental setup either in a mesocosm or in a controlled pond or lake system with a known number of individuals, all with sequenced reference mitogenomes; and (c) an in silico modelling approach using mitogenome data with controlled parameters of genetic diversity, allele frequencies, numbers of individuals present, as well as relative contribution of DNA from each individual. Such studies would provide crucial information for bridging the gap between eDNA and population genetic research, and while we at present operate with multiple unknown factors regarding the composition of eDNA samples, we illustrate here the power of eDNA as a means of variant exploration for population genetic inference.

As an unexpected advantage, the massive amounts of nontarget *E. affinis* DNA contributing to the sequence data simultaneously allowed us to explore mitochondrial variation with much higher coverage from a phylogenetically distant co‐inhabitant of the sampling site (Table S2), providing additional insight into the applicability of target capture for eDNA studies.

### 4.3 Nuclear capture

While the shortcomings of relying exclusively on mtDNA for population inferences have long been recognized (Ballard & Whitlock, 2004), eDNA researchers have focused on mtDNA due to its abundance as a multicopy marker, as well as to the large amount of reference data available in public databases. Nuclear target capture is largely unprecedented in eDNA studies, and with a lack of genomic reference data for most nonmodel organisms, this approach warrants extra caution. We have used here relevant available nuclear resources (i.e., genomes from *R. typus*, *Callorhinchus milli*, *Leucoraja erinacea*, *Scyliorhinus torazame* and *Chiloscyllium punctatum*) to ensure the best possible validation of the whale shark origin of eDNA sequences. Our study was not designed to explicitly estimate error rates, but a conservative estimate would suggest that about half of the raw whale shark variants found here represent sequencing and PCR errors. The coverage levels of the nuclear data retrieved for whale sharks were not sufficient for conducting in‐depth population genetic inferences, with only 22 variants passing the 10× depth filter (see also Figure S4 for raw mapping coverage). Furthermore, we are unable to exclude the possibility that some, if not all, of these variants simply represent sequencing errors, as the majority are represented by a single sequence deviating from the reference genome. However, this is an important first step and the first successful attempt at retrieving nuclear information from eDNA samples. Our probe design was optimized for ~60K probes, but it may well have been more beneficial with fewer probes in higher concentration, focusing on fewer loci, but with higher coverage per locus. We would also recommend incorporating more genomic resources of both closely and distantly related species during probe design, and to consider using stricter criteria regarding probe similarity (e.g., only including probes with <85% similarity to all other genomic resources), assuming that single species capture is the aim. Additionally, assuming that the quantity of target template could be the limiting factor, filtering more water could perhaps increase the efficiency of capture. Nevertheless, with higher sequencing output, and improved probe design and protocols, higher coverage may well be obtainable from environmental samples. This would enable researchers to shed light on population genomic variation through environmental sampling, rendering eDNA an increasingly useful noninvasive tool for population geneticists in the future.

Importantly, we would argue that the enormous amount of putative nuclear eDNA from *E. affinis* found in the sequencing data demonstrates this point. As we do not have a genome available for *E. affinis*, we cannot verify that nuclear reads stemmed from this species. However, the large amounts of mtDNA from *E. affinis* found here would suggest that *E. affinis* is the most likely source of nuclear reads. We were able to retain thousands of polymorphic variants with a minimum coverage of 20× (but sometimes as high as >1,000×). Analysis of nuDNA from environmental samples ultimately resemble a pooled sequencing approach (Sigsgaard et al., 2020), and it is noteworthy that such sequence coverage as obtained here in some respects fulfils the "best practices" requirements for pooled sequencing (Schlötterer et al., 2014). As compared to these "best practices," the problem nevertheless remains that it is difficult to know how many individuals have contributed DNA to the environmental samples and whether DNA contribution is reasonably balanced between individuals. Moreover, when using read counts and MAF to infer an approximated allele frequency spectrum, our data indicate that there is an abundance of rare alleles (an “L‐shape”; Figure 4). Reconstructing allele frequency spectra might serve as a preliminary test of the reliability of allele frequency estimates derived from environmental samples. In a relatively stable population, as would be expected to be the case for *E. affinis*, L‐shaped allele frequency spectra would be expected (Luikart et al., 1998), representing a high abundance of rare alleles. This was roughly in accordance with our results, although the L‐shape was not entirely clear‐cut, perhaps due partly to sequences from other species misidentified as *E. affinis* sequences. Multiple other scombrid fishes are known to occur at the sampling site, and if nuDNA sequences from some of these species were retained in the filtered reads and successfully mapped to the *Thunnus thynnus* genome, this may have obscured our view of allele frequencies.

Importantly, while tissue‐based population genetic studies can rely on genotyping data of single individuals to identify rare alleles, environmental data will have to rely on allele frequencies and allele counts within a sample. Some of the singleton allelic variants obtained will undoubtedly represent sequencing or PCR errors, but the influence of these can be mitigated by applying either an MAF filter or a minor allele count (MAC) filter. However, if sequencing depth is insufficient this will result in a simultaneous loss of true rare variants, and thereby render analyses based on rare variants unfeasible. Consequently, a high sequencing depth is needed to ensure multiple, independent hits to the minor allele for trustworthy inference. However, sequencing depth does not seem to influence MAF if the infrequent minor alleles (MAF < 0.05) are disregarded (Figure S5). Second, another safeguarding approach could be to use biological and/or technical replicates for decontamination of rare variants, as is the current standard in metabarcoding studies. When applying a replicate approach, rare variants could be removed, for example if they only appeared in one replicate. However, such an approach would increase the price of the study dramatically as additional capture reactions would be required, especially when performing custom probe design. Third, parallel analyses (using an identical number of PCR cycles and sequencing depth) of a prepared mock sample consisting of tissue‐derived DNA from several individuals with sequenced genomes would yield information on analysis‐specific error rates and how this can affect allele frequency estimation.

An enormous challenge for population genetic inference from environmental samples based on nuDNA will be the ability to discern between (a) novel genotypes from diverging populations or individuals of the target species, and (b) co‐occurring closely related species. Elucidating allele frequencies from environmental samples will be entirely dependent on the ability to confirm species‐level identification at each locus independently. If working in environments where multiple closely related species occur simultaneously, it would be worth considering designing probes in regions with diagnostic SNPs to safely infer species identification. However, population genetic inference would then only be possible if the flanking regions of the diagnostic SNP also hold population‐level information for the target species. We would argue that for proper probe design, researchers will need a reference genome of the target species as well as reference genomes from closely related species. To safely infer species‐level identification, it would be extremely useful to have multiple genomes of both target and nontarget species. As enough reference data are compiled, even novel genotypes from the target species could be determined based on eDNA, for example by presence of nuclear barcode gaps associated with each locus targeted.

### 4.4 Implications

This study demonstrates the wealth of genomic information on macroorganisms hidden in water samples and adds evidence to the increasing potential of eDNA as a population genetic tool. The relative read proportion of scombrid sequences from both the mitochondrial and the nuclear capture experiments proves that the capture approach is highly efficient in removing bacterial DNA, providing a massive advantage over shotgun sequencing for eukaryote monitoring (Stat et al., 2017). We show that whale shark DNA from entire mitochondrial genomes and multiple nuclear targets can be retrieved using target capture on water samples from an aggregation site. Sampling in the middle of groups of feeding and spawning fish was probably a close to ideal setting for testing the approach. However, any sort of aggregating behaviour that concentrates many individuals of a species of interest would probably be advantageous for this approach, and it is possible that even better results could be achieved in different settings, such as by sampling near marine mammals with aggregating haulout behaviour, seasonal shorebirds with feeding stopovers in coastal areas, fishes with schooling behaviour, mass migrating species or species present in plankton blooms.

We show that eDNA from water samples taken near aggregating individuals holds considerable potential in exploring both frequent and rare variants, which would otherwise require many individuals to be directly sampled. The emerging field of population genetics from environmental samples remains in its infancy, but as databases continue to expand to encompass complete mitochondria and nuclear data for more nonmodel organisms, we argue that the usefulness of this approach will increase substantially.

With cross‐capture previously being documented for nontarget organisms with up to 39% genetic divergence from target capture probes (Li et al., 2013), combined with the cross‐capture seen in this study, we argue that genomic target capture would furthermore hold promise as a multispecies approach in projects focused on entire organism groups (e.g., bony fish or mammals).

In conclusion, our study provides the first steps of baseline information on expected outcomes from population‐level target capture experiments on contemporary environmental samples. We show for two marine fish species, *R. typus* and *E. affinis*, that population genetic inference from entire mitogenomes and nuclear loci is indeed feasible with eDNA samples. Our study opens new frontiers in eDNA research, and holds great promise for future population genomic research on aggregating and spawning species in aquatic environments.

## CONFLICT OF INTEREST

The authors declare no conflict of interest.

## AUTHOR CONTRIBUTIONS

M.R.J. and P.F.T. conceived the ideas for the project. M.R.J., P.F.T., S.L. and M.M.H. designed the methodology. P.F.T., E.E.S., P.R.M. and S.S.B. carried out sampling in Qatar. M.R.J. conducted the laboratory work. M.R.J. analysed the data with guidance from S.L., A.M., E.E.S. and P.F.T. M.R.J. led the manuscript writing along with P.F.T and E.E.S. All authors contributed to the manuscript drafts and approved the final publication.

## DATA ACCESSIBILITY STATEMENT

Raw sequencing data are available on Dryad for both the mitochondrial and the nuclear capture. The data can be accessed via https://doi.org/10.5061/dryad.4mw6m9086. Enquiries regarding bioinformatics and pipelines should be directed to the corresponding authors.

## Supporting information

Appendix S1
