# Supplementary material for: Genome‐scale target capture of mitochondrial and nuclear environmental DNA from water samples
Source: Mol Ecol Resour. 2020 Nov 27;21(3):690–702. doi: 10.1111/1755-0998.13293 (PMC7983877; doi:10.1111/1755-0998.13293)
Supplement: Supplementary file 1 — Appendix S1 [file MEN-21-690-s001.docx]

Appendix S1

SUPPORTING INFORMATION

for

**Genome-scale target capture of mitochondrial and nuclear environmental DNA from water samples**

Mads Reinholdt Jensen^1*^, Eva Egelyng Sigsgaard^1^, Shenglin Liu^1^, Andrea Manica^2^, Steffen Sanvig Bach^3^, Michael Møller Hansen^1^, Peter Rask Møller^4^, Philip Francis Thomsen^1^.

1. *Department of Biology, Aarhus University, Ny Munkegade 116, Building 1540, DK-8000 Aarhus C, Denmark*
2. *Department of Zoology, University of Cambridge, Downing Street, Cambridge, CB2 3EJ, UK*
3. *Rambøll, Hannemanns Allé 53, DK-2300 Copenhagen S, Denmark*
4. *Natural History Museum of Denmark, University of Copenhagen, Universitetsparken 15, DK-2100 Copenhagen Ø, Denmark*

* Corresponding author. Tel.: +45 21 68 36 18, E-mail address: [mrj@bios.au.dk] (M. R. Jensen), ORCID: 0000-0001-8240-1083.

*Text A: Initial qPCR screening for whale shark DNA in samples:*

Samples were initially screened for whale shark eDNA with two sets of species-specific TaqMan qPCR systems (Thermo Fisher Scientific). We used the RhitypCB assay (Sigsgaard et al., 2017) for mitochondrial DNA content (cytochrome B). For nuclear DNA we designed a new assay, referred to as Rhityp_PGK1, using Primer3 (Untergasser et al., 2012). This assay targets 206 bp (incl. priming sites) of the Phosphoglycerate kinase 1 gene (PGK1), and consisted of forward primer Rhityp_PGK1_F (5’-GCCAGGGCTTCAATAAATCA-3’), reverse primer Rhityp_PGK1_R (5’-TGTCCAGCAATCAACCATGT-3’), and probe Rhityp_PGK1_P (5’-FAM-CAAGGCACCGCATTGTCCCA-BHQ1-3’). Separate qPCR replicates (duplicates for mitochondrial DNA, triplicates for nuclear DNA) were run with each marker, using for each 25 uL replicate reaction; 10 μL ddH2O, 10 μL TaqMan Environmental Master Mix, 1 μL forward primer (10 μM), 1 μL reverse primer (10 μM), 1 μL probe (2.5 μM), and 2 μL eDNA template. The thermocycler conditions were set with an initial 10 min preheat at 95 °C, followed by 55 cycles of 95 °C for 30 s and 60 °C for 60 s. Cycle threshold (Ct) values below 35 and 45 were used to confirm presence of mitochondrial and nuclear whale shark eDNA, respectively.

*Text B: Designing nuclear DNA capture probes for whale shark:*

A bait set of 40,000-60,000 probes (cat. no. 300348) of 80 bp in length and with dual-tiling was designed in collaboration with the company myBaits (Arbor Biosciences). Using a custom script written in AWK and R, we extracted non-repetitive intron and exon coordinates from the genome. The coordinates constituted ~580 Mb of sequence, and since we intended to limit the probes to span 2-3 Mb of the genome, we focused the probe design to fragments of >1500 bp in length. Overlapping regions between intron and exon coordinates were inspected using BEDTools (Quinlan & Hall, 2010), but were finally ignored due to their very limited extent (~0.18 %). A list of exon and intron coordinates (~0.8 Mb of exons and ~5.3 Mb of introns) was then sent to myBaits, who performed a probe performance filtering, keeping only baits that passed their stringent BLAST filtering. No probes from repetitive regions were included, and all probes with slight affinity to the mitochondrial genome were removed. Exon baits were filtered to only include baits with a GC-content of 34-51 % and a ΔG-value > -3. Intron baits were filtered to include baits with a GC-content of 33-52 % and a ΔG-value > -3. As the purpose was a probe design that was as specific to whale sharks as possible, the suggested probe design was subsequently blasted against four other chondrichthyan genomes available on NCBI (Australian ghostshark (*Callorhinchus milli*), little skate (*Leucoraja erinacea*), cloudy catshark (*Scyliorhinus torazame*), and brownbanded bamboo shark (*Chiloscyllium punctatum*)) to remove probes with equal affinity to other species of sharks, and thereby allow better discrimination at the species level. We removed all probes with 100 % matches to one or more of these other cartilaginous fishes. We also blasted the probes against the GenBank Nucleotide database, and removed probes with hit lengths > 40 bp. The final, optimized probe set consisted of 6,008 exon probes and 53,933 intron probes (59,941 probes total).

*Text C: Capture efficiency of nuclear probes:*

Altogether, the 59,941 probes targeted 26,425 genomic regions (2,822 exonic and 23,618 intronic regions), a total of 3,484,519 bp (355,950 exonic bp and 3,130,231 intronic bp) of the whale shark genome, with a trivial overlap of 1,662 bp between introns and exons. Based on the 48,433 reads that mapped to the whale shark genome, we found that a total of 19,129 genomic regions of 5,669,877 bp were covered by the sequencing reads of whale shark origin. From these, 2,146,660 bp overlap with the targeted regions, which amounts to 37.9 % of the sequenced regions and 61.6 % of the targeted regions. In total, 1,915 targeted exonic regions (67.9%) with 215,258 bp (60.5%) were captured by 911 sequenced regions. Likewise, 16,380 targeted intronic regions (69.4%) with 1,932,427 bp (61.7%) were captured by 7,712 sequenced regions. Overall, 18,285 targeted regions (69.2%) with 2,146,660 bp (61.6%) were captured by 8,618 sequenced regions.

**Figure S1:** Overview of read lengths of the 16,474 filtered mitochondrial reads that were mapped to the whale shark mitochondrial genome (acc. no. NC_023455) as putative whale shark reads.


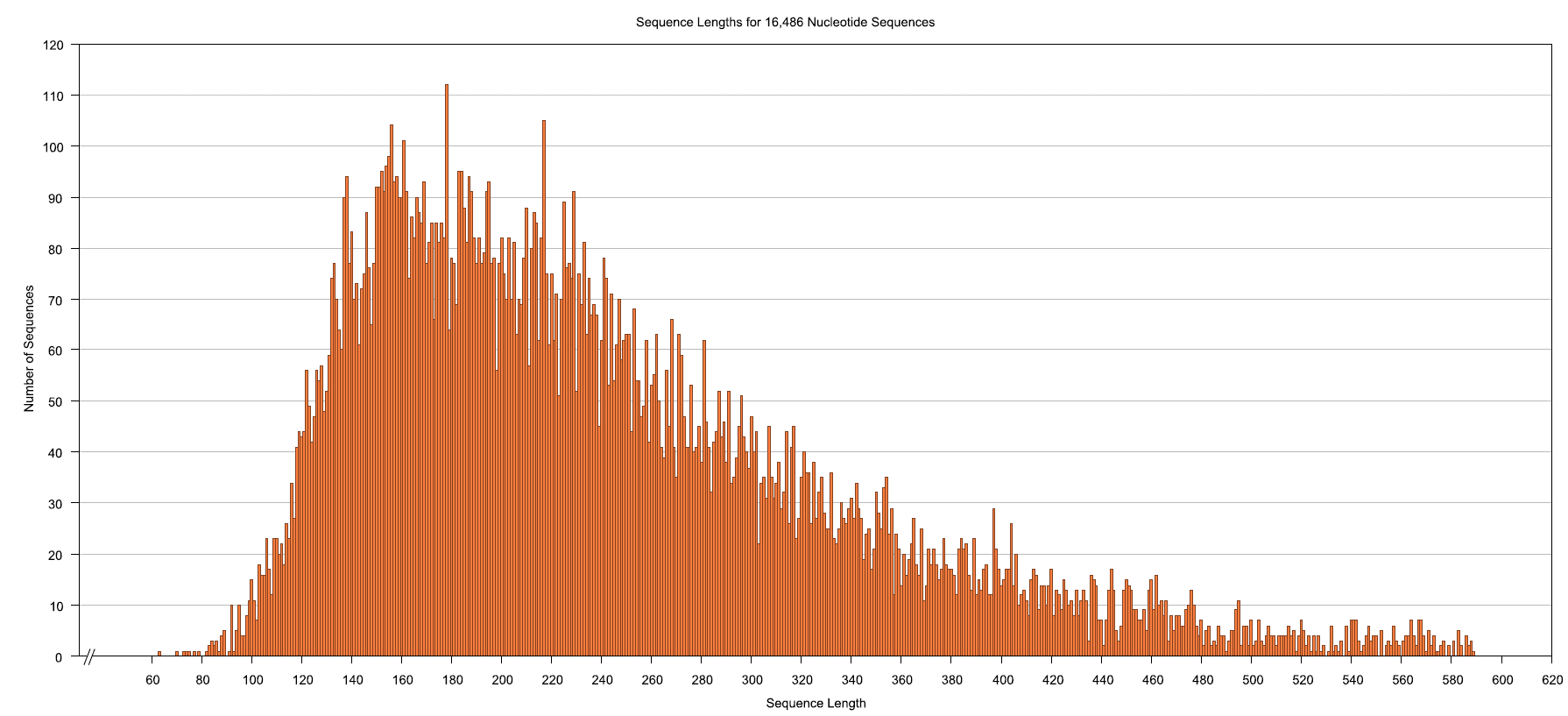


**Figure S2**: Scatterplot of similarity plotted against relative coverage of mitochondrial reads with *E*. *affinis* as their best blast hit, as presented in Fig. 2. Each dot represents a single bp in the mitochondrial genome. Red line depicts a highly significant linear regression (y = 0.31x-0.032, Adjusted R^2^ = 0.058, p < 0.001) with 95 % confidence intervals.


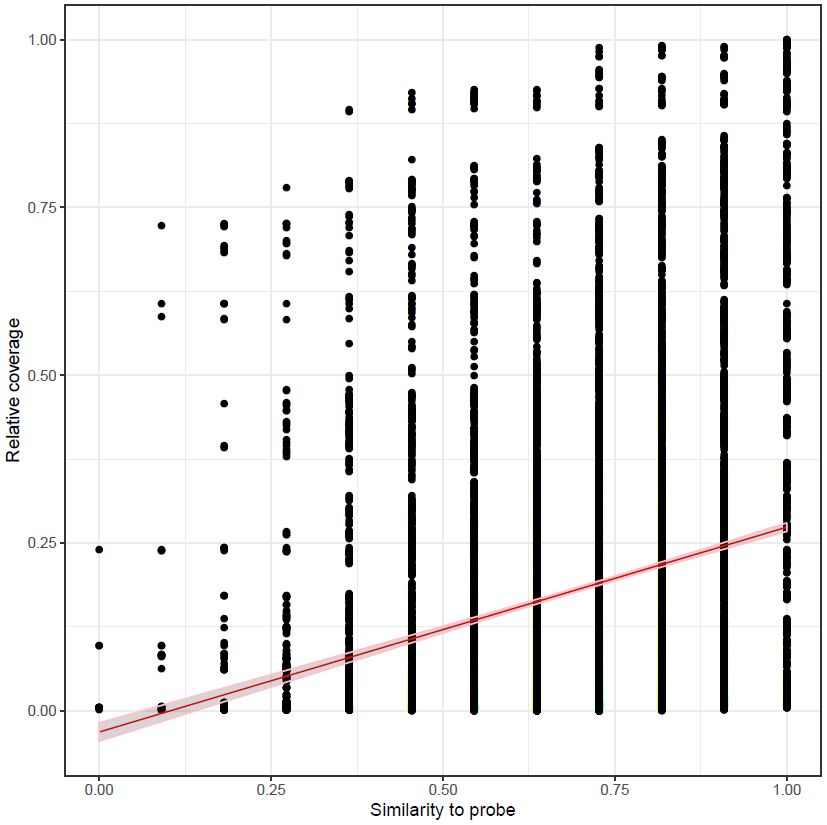


**Figure S3:** Overview of read lengths of the 48,433 filtered nuclear reads that were mapped to the whale shark nuclear genome (acc. no. GCA_001642345.2) as putative whale shark reads.


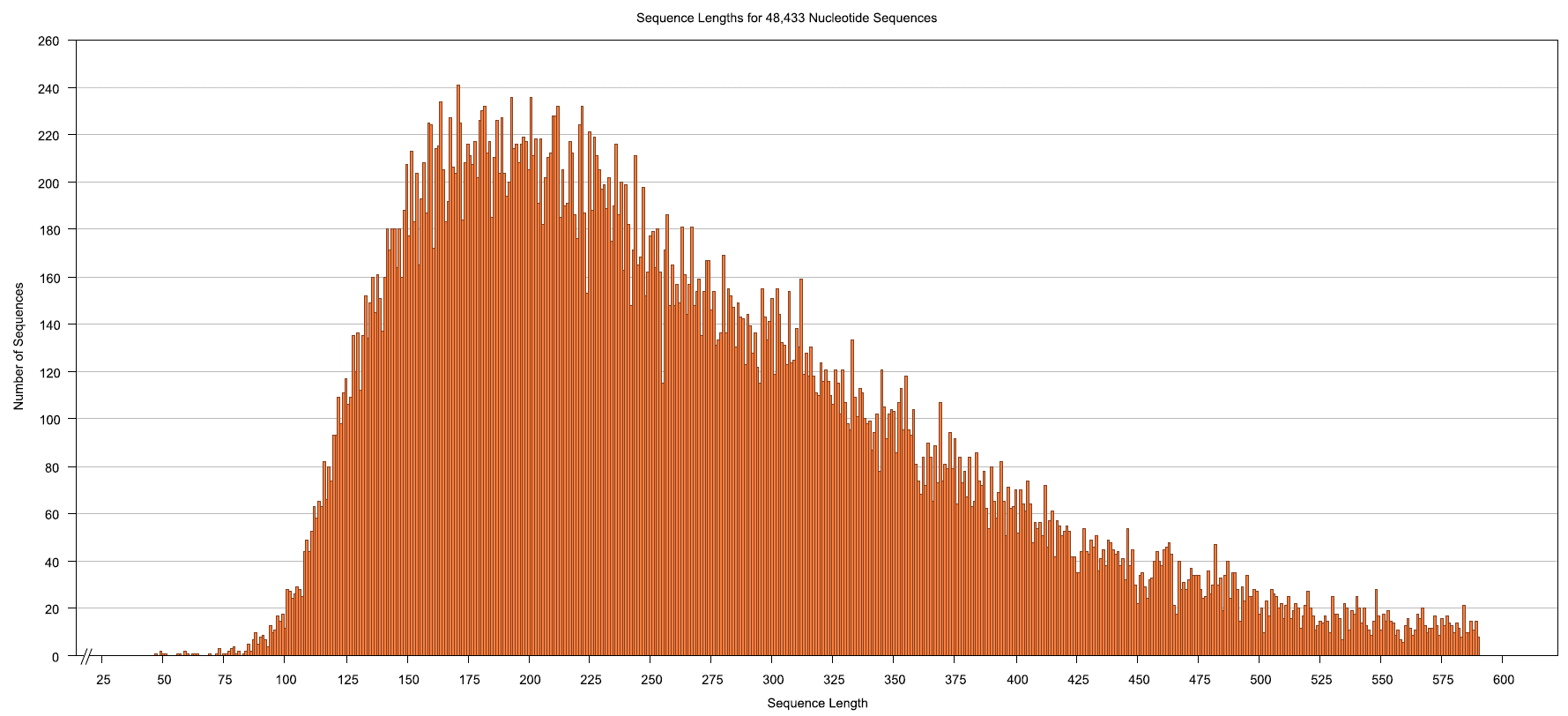


**Figure S4**: Coverage distribution plot of mapped reads from the nuclear DNA capture to *R*. *typus* acc. no. GCA_001642345.2 (Fig. S4A) and *T*. *thynnus* acc. no. GCA_003231725.1 (Fig. S4B).


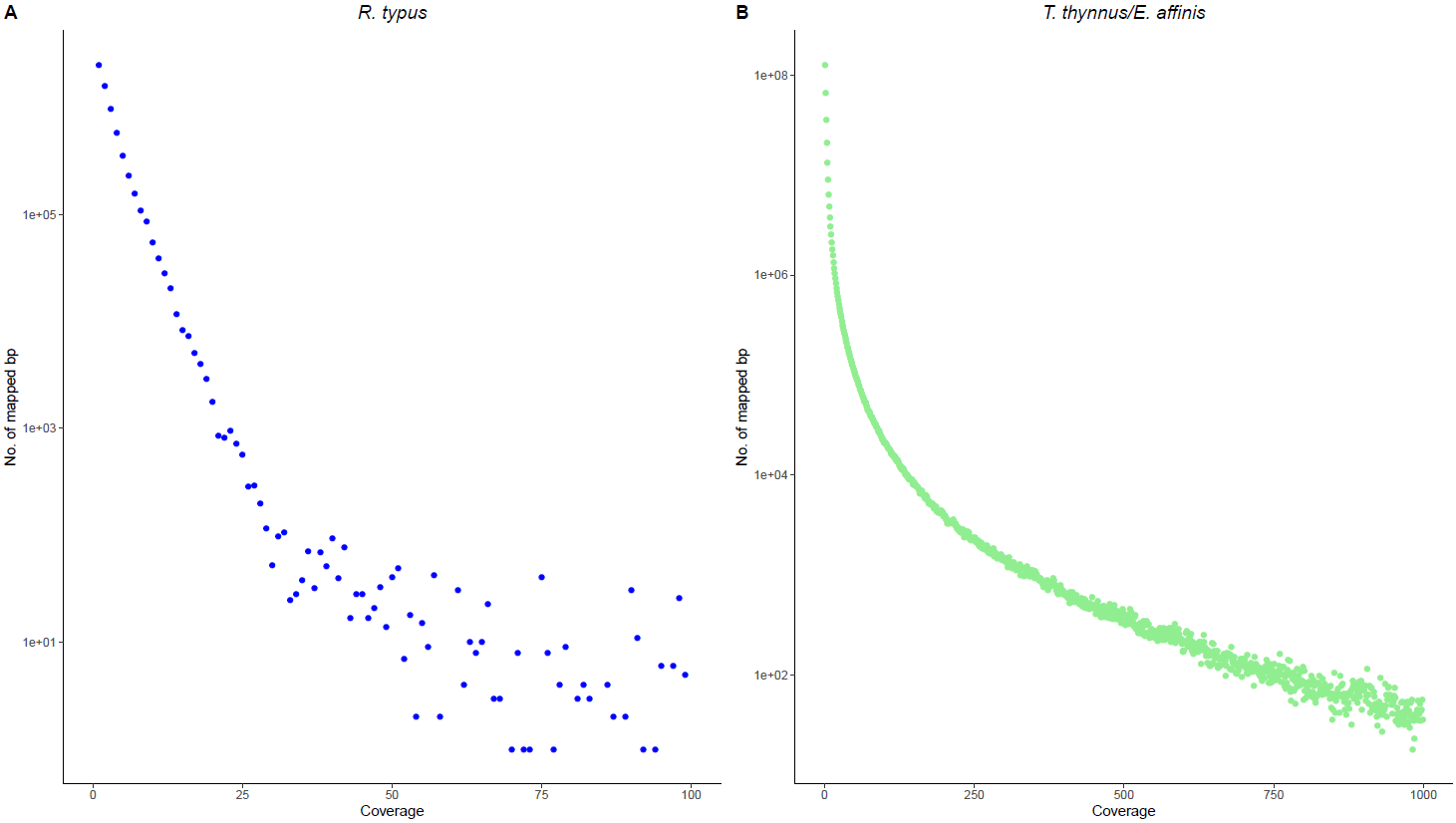


**Figure S5:** Scatterplot of depth (x-axis) against MAF (y-axis) from polymorphic nuclear variants mapping to *T. thynnus*, with a minimum depth of 20. A) and C) represent all variants, whereas B) and D) represent only variants with MAF ≥ 0.05. In C) and D) we applied a rank-based inverse normal transformation (INT) on both axes to accommodate normal distribution and to avoid violating linear regression assumptions on normality. Red lines depict highly significant linear regression in C) (y = -0.25x+0.00058, Adjusted R^2^ = 0.06328, p < 0.001), and non-significant linear regression in D) (y= 0.36x+0.0019, Adjusted R^2^ = -0.0028, p = 0.362). Note that a variant with depth = 20 (our filter) does not enable a MAF < 0.05 (lack of data points on A) bottom left), and that high depth results in many low MAF data points (bottom right A)) that are expected to contain a higher degree of sequencing errors.


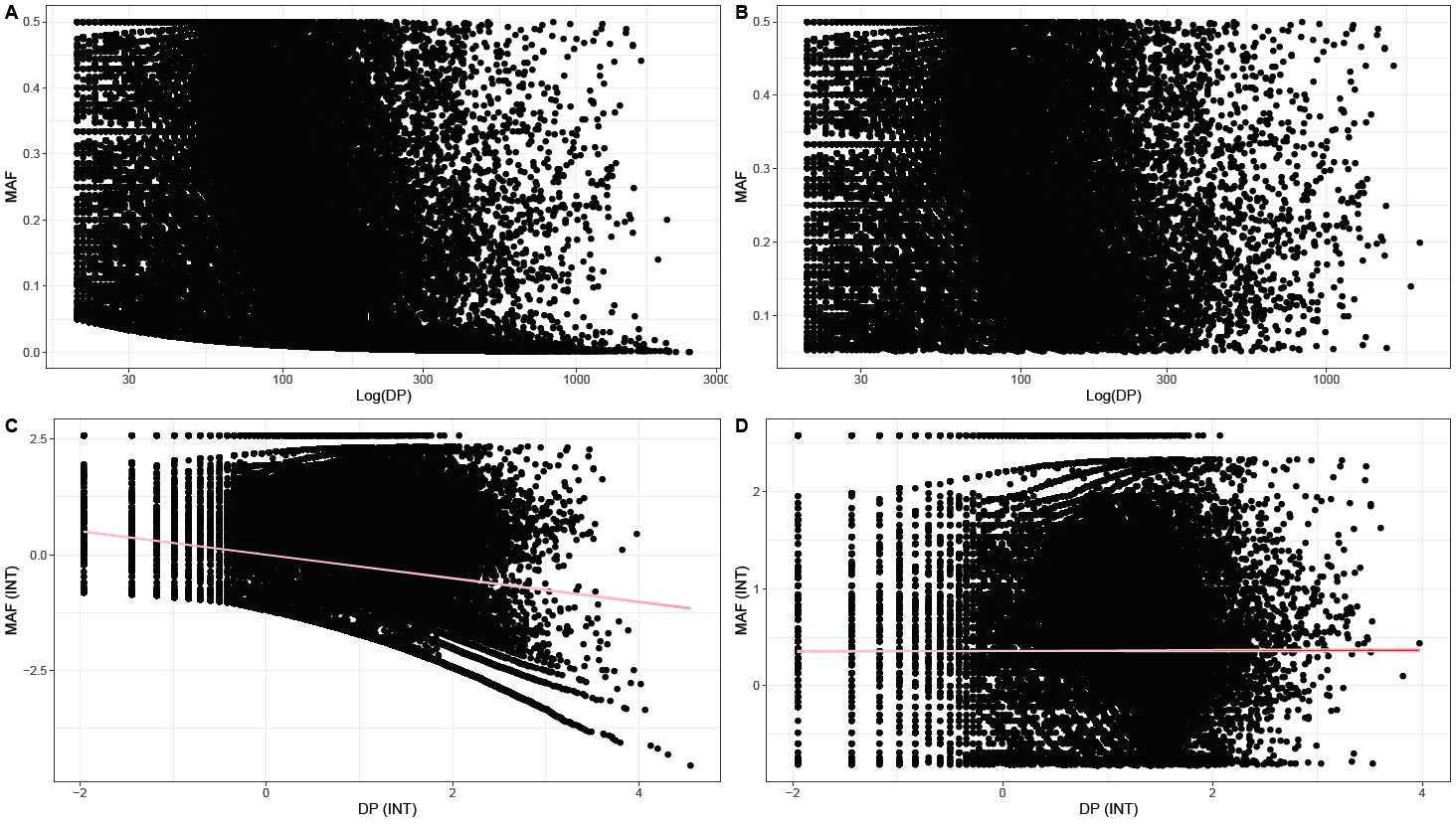


**Table S1:** Representation of mitochondrial reads having best or equally best match to a given group of organisms. Only reads with 100 % similarity, a minimum of 150 bp length and a minimum of 100 blast hits are included in this table. It is important to note that the list of species contributing only represents 100 % hits in GenBank, and that there could be multiple hits with 100 % match. A denoted species does therefore not necessarily mean that this species is present in these waters, nor that this species is correctly identified, as we did not perform a thorough taxonomic investigation of all reads. This list is simply to illustrate the magnitude of hits towards whale sharks and scombrids in relation to what else is present in the data when applying a target capture protocol.

| **Table S1** | | | | |
| --- | --- | --- | --- | --- |
| Order | Family | Order hits | Family hits | Species contributing |
| Orectolobiformes | | 21,178 |  |  |
|  | Rhincodontidae |  | 21,178 | *Rhincodon typus* |
| Perciformes | | 1,927 |  |  |
|  | Apogonidae |  | 172 | *Sphaeramia orbicularis* |
|  | Echeneidae |  | 105 | *Echeneis naucrates* |
|  | Percidae |  | 1,440 | *Sander lucioperca* |
|  | Sparidae |  | 210 | *Sparus aurata* |
| Scombriformes | | 7,604,167 |  |  |
|  | Scombridae |  | 7,604,167 | *Auxis rochei*, *Auxis thazard*, *Euthynnus affinis*, *Euthynnus alletteratus*, *Katsuwonus pelamis*, *Sarda orientalis*, *Scomber scombrus*, *Scomberomorus commerson*, *Thunnus orientalis* |
| Oscillatoriales | | 2,902 |  |  |
|  | Microcoleaceae |  | 2,902 | *Trichodesmium erythraeum* IMS101 |
| Synechococcales | | 468 |  |  |
|  | Synechococcaceae |  | 468 | *Synechococcus* sp. KORDI-52, *Synechococcus* sp. WH 8109 |
| Pelagibacterales | | 1,735 |  |  |
|  | Pelagibacteraceae |  | 1,735 | *Pelagibacter* sp. FZCC0015, *Pelagibacter* sp. RS39, Alpha proteobacterium HIMB59 |
| ”Uncultured” | | 604 |  |  |
|  | Marine organism |  | 296 |  |
|  | Bacteria |  | 308 |  |

**Table S2:** Overview of mitochondrial variants when mapping putative *E*. *affinis* reads to the *E*. *affinis* mitogenome (acc. no. NC_025934) with a 5 % minor allele frequency requirement. Loc: location of the variant. Mut type: type of mutation, either transition (TI), transversion (TV), or insertion (INS). AA change: amino acid change. CDS position: location of the variant within coding sequence. Cov: coverage. Prot effect: Protein effect, either substitution (Sub) or none. KV: known variant from sequenced tissue samples of *E*. *affinis* deposited in GenBank (+) or unknown variant (-).

| **Table S2** | | | | | | | | | | | |
| --- | --- | --- | --- | --- | --- | --- | --- | --- | --- | --- | --- |
| Variant no | Loc | Gene | Nucl. change | Mut type | AA change | CDS position | Codon change | Cov | Prot effect | Allele freq (%) | KV* |
| 1 | 2,784 | trnL(AAA) | C 🡪 T | TI |  |  |  | 160,592 |  | 99.9 | + |
| 2 | 3,363 | ND1 | G 🡪 A | TI |  | 507 | CAG 🡪 CAA | 34,124 | None | 7.5 | - |
| 3 | 3,522 | ND1 | G 🡪 A | TI |  | 666 | GCG 🡪 GCA | 72,806 | None | 6.0 | - |
| 4 | 3,651 | ND1 | G 🡪 A | TI |  | 795 | CTG 🡪 CTA | 29,177 | None | 26.4 | + |
| 5 | 4,182 | ND2 | G 🡪 A | TI |  | 138 | CAG 🡪 CAA | 45,475 | None | 35.5 | + |
| 6 | 4,248 | ND2 | T 🡪 C | TI |  | 204 | GCT 🡪 GCC | 27,944 | None | 11.1 | - |
| 7 | 4,674 | ND2 | A 🡪 G | TI |  | 630 | ATA 🡪 ATG | 794 | None | 37.9 | + |
| 8 | 6,037 | COI | G 🡪 A | TI |  | 558 | TGG 🡪 TGA | 6,262 | None | 6.9 | + |
| 9 | 6,196 | COI | C 🡪 T | TI |  | 717 | GGC 🡪 GGT | 39,144 | None | 8.3 | - |
| 10 | 8,568 | ATP6 | G 🡪 A | TI |  | 459 | CGG 🡪 CGA | 86,235 | None | 10.3 | - |
| 11 | 8,772 | ATP6 | C 🡪 T | TI |  | 663 | CTC 🡪 CTT | 143,465 | None | 6.2 | - |
| 12 | 9,176 | COIII | G 🡪 A | TI |  | 384 | GAG 🡪 GAA | 76,250 | None | 99.9 | + |
| 13 | 9,239 | COIII | C 🡪 T | TI |  | 447 | CAC 🡪 CAT | 50,561 | None | 99.9 | + |
| 14 | 9,925 | ND3 | A 🡪 G | TI | T 🡪 A | 277 | ACC 🡪 GCC | 1,254 | Sub | 99.5 | + |
| 15 | 10,749 | ND4 | G 🡪 A | TI |  | 393 | CTG 🡪 CTA | 10,405 | None | 6.6 | - |
| 16 | 10,977 | ND4 | G 🡪 A | TI |  | 621 | AAG 🡪 AAA | 49,979 | None | 99.9 | + |
| 17 | 10,983 | ND4 | G 🡪 A | TI |  | 627 | CCG 🡪 CCA | 51,752 | None | 99.9 | + |
| 18 | 12,483 | ND5 | C 🡪 T | TI |  | 531 | GTC 🡪 GTT | 1,038 | None | 6.6 | - |
| 19 | 12,837 | ND5 | T 🡪 C | TI |  | 885 | GCT 🡪 GCC | 49,532 | None | 9.6 | - |
| 20 | 12,903 | ND5 | G 🡪 A | TI |  | 951 | GGG 🡪 GGA | 71,484 | None | 8.0 | - |
| 21 | 13,134 | ND5 | G 🡪 A | TI |  | 1182 | GGG 🡪 GGA | 76,042 | None | 7.1 | - |
| 22 | 13,365 | ND5 | C 🡪 T | TI |  | 1413 | GGC 🡪 GGT | 22,766 | None | 11.1 | - |
| 23 | 14,208 | ND6 | C 🡪 G | TV |  | 102 | GTG 🡪 GTC | 2,299 | None | 99.7 | + |
| 24 | 14,226 | ND6 | A 🡪 G | TI |  | 84 | GCT 🡪 GCC | 2,344 | None | 99.6 | + |
| 25 | 15,393 | Cyt B | G 🡪 A | TI |  | 1011 | TGG 🡪 TGA | 43,064 | None | 98.0 | + |
| 26 | 15,750 | D-loop | A 🡪 G | TI |  |  |  | 429 |  | 21.2 | + |
| 27 | 15,751 | D-loop | C 🡪 T | TI |  |  |  | 421 |  | 27.8 | + |
| 28 | 15,773 | D-loop | T 🡪 C | TI |  |  |  | 321 |  | 10.3 | + |
| 29 | 16,222 | D-loop | G 🡪 A | TI |  |  |  | 341 |  | 8.2 | - |
| 30 | 16,232 | D-loop | G 🡪 A | TI |  |  |  | 347 |  | 85.9 | + |
| 31 | 16,274 | D-loop | T 🡪 C | TI |  |  |  | 365 |  | 6.0 | - |

*References cited in Supporting Information:*

Quinlan, A. R., & Hall, I. M. (2010). BEDTools: A flexible suite of utilities for comparing genomic features. *Bioinformatics*, *26*(6), 841–842. https://doi.org/10.1093/bioinformatics/btq033

Sigsgaard, E. E., Nielsen, I. B., Bach, S. S., Lorenzen, E. D., Robinson, D. P., Knudsen, S. W., Pedersen, M. W., Jaidah, M. A., Orlando, L., Willerslev, E., Møller, P. R., & Thomsen, P. F. (2017). Population characteristics of a large whale shark aggregation inferred from seawater environmental DNA. *Nature Ecology & Evolution*, *1*(1), 0004. https://doi.org/10.1038/s41559-016-0004

Untergasser, A., Cutcutache, I., Koressaar, T., Ye, J., Faircloth, B. C., Remm, M., & Rozen, S. G. (2012). Primer3—New capabilities and interfaces. *Nucleic Acids Research*, *40*(15), e115–e115. https://doi.org/10.1093/nar/gks596
